# Measuring changes in hydrolysis concept of students taught by inquiry model: stacking and racking analysis techniques in Rasch model

**DOI:** 10.1016/j.heliyon.2022.e09126

**Published:** 2022-03-17

**Authors:** Lukman Abdul Rauf Laliyo, Bambang Sumintono, Citra Panigoro

**Affiliations:** a Department of Chemistry, Faculty of Mathematics and Natural Sciences, Universitas Negeri Gorontalo, Gorontalo, 96128, Indonesia; b Faculty of Education, Universitas Islam Internasional Indonesia, 16416, Indonesia; c Department of Aquatic Resource Management, Faculty of Fisheries and Marine Science, Universitas Negeri Gorontalo, 96128, Indonesia

**Keywords:** Stacking, Racking, Rasch model, Hydrolysis conceptual changes, Inquiry model

## Abstract

This research aimed to employ stacking and racking analysis techniques in the Rasch model to measure the hydrolysis conceptual changes of students taught by the process-oriented guided inquiry learning (POGIL) model in the context of socio-scientific issues (SSI) with the pretest-posttest control group design. Such techniques were based on a person- and item-centered statistic to determine how students and items changed during interventions. Eleventh-grade students in one of the top-ranked senior high schools in the eastern part of Indonesia were involved as the participants. They provided written responses (pre- and post-test) to 15 three-tier multiple-choice items. Their responses were assessed through a rubric that combines diagnostic measurement and certainty of response index. Moreover, the data were analyzed following the Rasch Partial Credit Model, using the WINSTEPS 4.5.5 software. The results suggested that students in the experimental group taught by the POGIL approach in the SSI context had better positive conceptual changes than those in the control class learning with a conventional approach. Along with the intervention effect, in certain cases, it was found that positive conceptual changes were possibly due to student guessing, which happened to be correct (lucky guess), and cheating. In other cases, students who experienced negative conceptual changes may respond incorrectly due to carelessness, the boredom of problem-solving, or misconception. Such findings have also proven that some students tend to give specific responses after the intervention in certain items, indicating that not all students fit the intervention. Besides, stacking and racking analyses are highly significant in detailing every change in students’ abilities, item difficulty levels, and learning progress.

## Highlights

• Raw scores have a bias in a conventional psychometric measurement
• Stacking and racking measure students' ability and item difficulty level changes
• The learning process in socio-scientific issues improves students' understanding
• Misconceptions influence the negative values of students' pre-and post-test

## 1 Introduction

Central to defining the quality of pedagogical innovation in science classes is conceptual changes. The changes refer to how ideas or conceptions the students understand according to their ways of thinking [1, 2] become scientifically accurate [3]. It is because such ideas generally comprise misconceptions [4, 5, 6, 7], are not in accordance with scientific concepts [8, 9], tend to be resistant [10], changeable and varied [11], so that they should be improved if the correct conceptual understanding is to be taught [12, 13].

Some studies have been conducted on learning innovation testing to form an accurate and scientific conceptual understanding of the students, e.g., inquiry-based learning. This model presents conceptual conflicts and participatory experiments to facilitate conceptual changes [14, 15, 16]. Conceptual understanding-based learning involves various strategies in identifying and analyzing students' comprehension so that the investigation process can be designed to lead them to a more accurate and scientific conception [16, 17]. This research relied on a quasi-experimental design that assessed students’ pre-test and post-test, evaluated the changes in performances for testing significant differences. This type of testing informs the researcher about the presence of an effect, but does not provide detailed information on the level and trait of the changes [18]. What if the researcher is willing to compare the extent to which the pre- and post-test change (differences in learning outcomes) and interpret the changes (the reasoning why those changes occur) in terms of content? This is a core question regarding the changes in some latent traits or changes in traits measured after the intervention. In most studies, interpreting the changes in pre-test and post-test tends to be limited to identifying whether or not an effect prevails.

Pre- and post-test changes should be given in detail regarding the students’ understanding ability and item difficulty levels. However, this has not been much revealed due to the limitations of its measurement techniques and analyses and has not been the main focus in chemistry education research to date. One reason for this issue is the debate in the psychometric community regarding the ability to measure changes accurately [18]. This debate questions the use of raw scores in the conventional psychometric analysis, which largely contains measurement biases [19], as follows: 1) the difference in pre- and post-test scores will be negatively correlated with the pre-test score, especially for students with low pre-test scores [18,20]; 2) the difference in pre- and post-test scores shows low test reliability [21]; 3) low measurement properties due to different scales [22].

Raw scores are not final data, so that they do not have a great deal of information for drawing conclusions [23, 24]. Around the 1950s, Dr. Georg Rasch, a mathematician from Denmark, introduced the formulation of the Rasch measurement model [24]. The model has been widely applied to analyze various types of data, e.g., dichotomous, polytomous, multi-rating, and multi-rater data. In the mid-2000s, the Rasch model was used as a probabilistic-based psychometric measurement that went beyond the use of raw scores [25, 26], and was used to overcome the limitations of conventional psychometric measurement [19, 27]. Its analyses, including item fit, PCA (Principal Component Analysis), and Wright map, are commonly used for international test analyses, namely TIMSS and PISA [28].

In chemistry education research, the Rasch model has been relied on to evaluate learning understanding and progress [29], to diagnose students' preconceptions [1], misconceptions [13, 30, 31, 32], link the measurement of content knowledge with pedagogical content knowledge [33], and investigate item difficulty patterns [13, 34]. Even so, studies on the Rasch model to reveal the chemistry conceptual changes in students’ understanding and item difficulty levels are relatively hard to find as of today. The present study aims to employ stacking and racking analysis techniques in the Rasch model to measure the hydrolysis conceptual changes of students taught by the POGIL approach in the context of SSI and students who learn conventionally. Such techniques are based on a person- and item-centered statistic to estimate how students and items change during the intervention.

POGIL is a student-centered learning strategy that teaches content or process skills. The philosophical foundation of POGIL is the involvement of an interactive process of careful thinking, discussing ideas, perfecting understanding, practicing skills, reflecting progress, and evaluating performances [35]. POGIL is able to lead the process of designing a participatory experiment that presents a conceptual conflict as a strategy to encourage students to form an accurate concept [14]. Therefore, POGIL intervention is more likely to be potential in driving epistemological understanding and reasoning [36], making students have opportunities to change their conceptions to be more accurate and scientific [16]. Nevertheless, it is also worth noting that some students potentially have misconceptions resistant to changes [3].

SSI functions as a learning context through the integration of social problems that students are familiar with. It also has a conceptual connection with salt hydrolysis [37, 38], and its resolution requires many perspectives [39], including the dimension of moral and ethical evaluation of students [40]. The SSI context is a socio-scientific phenomenon that the students should explain based on their conceptual viewpoints. It encourages them to actively get involved in grasping problems [41], developing and utilizing their knowledge [42], improving their critical thinking [43], and being able to scientifically describe the discussed socio-scientific phenomenon [36, 44, 45]. For such reasons, the integration of SSI can build up students' scientific literacy [39, 46, 47]. In the end, this integration enables the learning process to be more significant in enhancing students’ understanding [45, 48]. Besides, they are skilled in negotiating the social aspect of the studied phenomenon [49, 50]. For instance, the issues of global warming, climate change, and pollution [36].

Salt hydrolysis is a learning topic in high school that is strongly related to SSI. Students with a good understanding of hydrolysis will manage to clarify scientifically why detergents, bleaching agents (NaOCl), and fertilizers can pollute the environment. Despite this, the linkage of this issue as the problem in learning hydrolysis is rarely carried out. The learning process is more emphasized on mastering theoretical concepts [36]. As a consequence, students find it challenging to use their hydrolysis understanding to explain socio-scientific phenomena around them [37]. This challenge is on account of their misconceptions regarding acid-base reaction [51], making them unable to elaborate the concept of salt hydrolysis [52] and determine acid and base strength [53]. In addition, they are struggling with correctly explaining the dissolving process and the reaction of ionic compounds with water, writing down chemical equations, and having different interpretations of the dissolving process mentioned earlier [54]. On this ground, it is essential to reveal how the hydrolysis concept changes if intervened with the POGIL approach in the SSI context, through the following specific questions: (1) is there a significant hydrolysis conceptual change of the students after the learning process in experimental and control groups? (2) if compared, how is the hydrolysis conceptual change through the intervention of POGIL in the SSI context and conventional learning? (3) in addition to intervention, is there any other factor that also contributes to the students' hydrolysis conceptual changes?

## 2 Method of study

This study relied on a quantitative approach with a quasi-experimental and pretest-posttest control group design [55] by comparing the extent to which the hydrolysis concept changes after the intervention. Researchers carried out the learning process for 12 meetings, gave tests, and collected data on the results of the intervention and measurement.

The changes of students and items were analyzed using the stacking and racking techniques in the Rasch model [56]. As standard techniques, racking and stacking were introduced by Benjamin Wright to measure the extent to which conceptual understanding of students and items change before and after interventions [57]. The referred changes are cases (item and student levels) caused by the learning intervention and can be diagnosed based on the estimated changes.

In regards to students' understanding, the measurement was to identify students who had specific hydrolysis conceptual changes in responding to the learning intervention. In terms of items, the measurement was done to identify which items had special characteristics and been understood by students differently during the learning intervention [57]. Thus, the scientific inquiry approach might not be suitable for some students, or some items might be too hard after the intervention. This insightful information is immensely helpful for researchers and education practitioners, especially in evaluating the weaknesses of pedagogical innovations being applied and devising learning strategies that meet students’ needs in learning [58].

### 2.1 Participants

Eleventh-grade students aged 16–17 years in one of the senior high schools in the eastern part of Indonesia were involved as the sample. This top-ranked school gets an “A” accreditation (excellent) from the National Accreditation Board for High School. The sample was determined by convenience sampling in six randomly assigned classes. Three classes (N = 97) were experimental groups that applied the POGIL model in the SSI context. The other three classes (N = 93), as control groups, applied conventional learning without the SSI context. The same teacher taught these classes following the Curriculum 2013 of Chemistry Subject (revised in 2016). There was no special classroom for learning the concept of hydrolysis, i.e., taking up the regular learning process at school. Before learning the hydrolysis concept, the students had previously learned the concept of acid and base to understand the concept of salt hydrolysis way better. Research permission was obtained from the government and school administrators. In accordance with principles of research ethics, research purpose and procedures were informed to all the students being involved and that they were voluntarily participating. Additionally, their information is confidential and only used for science development [59].

### 2.2 Learning implementation

Students in the experimental group studied employing the process-oriented guided inquiry learning (POGIL) in the SSI context [35]. Meanwhile, in the control class, the learning process was performed conventionally; the teacher facilitated learning initiatives. The learning process focused more on content mastery and problem-solving practice. Applying the POGIL model in the SSI context highlights teacher assistance to guide the students to prepare their conceptual understanding based on epistemological reasoning they get from experiments, discussions, and collaborations [49, 60]. Researchers carried out the learning process for eight weeks to apply the intervention to the sample, gave tests, collected data on the results of the intervention and measurement. The first three weeks were the preparation stages when researchers and the teacher shared perceptions, and asked the teacher to perform a learning simulation under the scenario, including different assistance techniques in leading the students to conduct experiments, and to ask analytical questions. The pre-test was carried out in the third week. Further, the learning implementation was done for four weeks, and the post-test was executed in the eighth week.

The learning stages with POGIL in the SSI context consist of orientation, exploration, concept formation, application, and closing. During the orientation stage, the teacher presented familiar contextual phenomena related to the concept of hydrolysis. The teacher asked initial questions to provoke curiosity and arouse motivation and interest of the students. While watching the video, had the students responded and explained the relationship between the phenomena and acids and bases, hydrolysis, and buffers. In the exploration stage, the teacher developed analytical questions with data, images, and multiple video clips to give perspectives on learning objectives and to delve into the concept that had been and would be learned. Next, the teacher assisted the students in doing experiments guided by a worksheet, and at the same time, asked analytical questions to lead them and strengthen their conceptual understanding. In the concept formation stage, the teacher asked students to build their conceptual understanding based on the exploration results, accompanied by critical and fundamental questions to guide students in building a conceptual understanding of the salt hydrolysis and buffer solution.

Following the formation stage was the application stage when the teacher presented contextual problems in the SSI context, particularly those comprising social problems in society, that closely linked with the understanding of salt hydrolysis and buffer solution concepts. Such problems included 1) the use of bleaching agents (detergents), 2) the functions of alum KAl(SO₄)₂·12H₂O for water purification, 3) the harmful effects of detergent waste, 4) the beneficial and harmful effects of artificial fertilizer (NH_4_)_2_SO_4_ for soil fertility, and 5) the harmful effects of monosodium glutamate (MSG) for health. In this stage, the teacher guided the students through collaborative discussions and critical questions, intending to give them perspectives on SSI phenomena and encourage them to collect information and do experiments following student activity sheets. Thereupon, the students had presentation and discussion sessions, during which they reported their experiment results and drew conclusions [61, 62]. The teacher asked them to describe the possible problems and solutions from their understanding of the studied concepts. This enabled the students to form their conceptual understanding that is closely related to contexts; the learning process was from contextual to abstract [37, 63]**.** From such a condition, the teacher led the students to apply their knowledge in different contexts and situations and solve problems. The final stage was closing or teacher assistance in guiding the students to explain the conclusion and reflection on the learning process as the end of the learning activities.

### 2.3 Instrument

Table 1 displays 15 items of diagnostic three-tier multiple choice test to measure students’ hydrolysis conceptual understanding. The test was constructed following the Competence Standard of 2013 Chemistry Curriculum of Senior High School under Regulation of the Minister of Education and Culture of the Republic of Indonesia Number 37 of 2018. The procedures of developing the instrument followed the recommendation by [64, 65, 66].

**Table 1:** Conceptual map of hydrolysis concept understanding [67].

Problem Context	Item	Conceptual Understanding	Ability Level	
Bleaching agents are formed of weak acid HOCl and strong base NaOH. Sodium hypochlorite salt (NaOCl) is reactive and dissolves the dye. In the water, the ion ${\text{OCl}}^{-}$ will be hydrolyzed to HOCl and ${\text{OH}}^{-}$^-^	1	Balancing the salt (NaOCl) hydrolysis reaction in the water	2	Level 3: Students are able to calculate the pH of the hydrolyzed salt solution. Level 2: Students are able to determine the hydrolysis reaction from different types of salt Level 1: Students are able to analyze the properties of the hydrolyzed salt
	2	Stating the partial hydrolysis reaction: $\text{NaOCl}\to \;{\text{Na}}^{+}+\;{\text{OCl}}^{-}$	2	
	3	Determining corrosive alkali of sodium hypochlorite salt (NaOCl)	1	
	4	Calculating the pH of hydrolysis of sodium hypochlorite salt (NaOCl) with NaOCl = 0.1 M; Ka = 10^−5^)	3	
	5	Determining the property of NaOCl, in the reaction: ${\text{OCl}}^{-}+\;{\text{H}}_{2}\text{O}\to \text{HOCl}+\;{\text{OH}}^{-}$	2	
	6	Calculating the pH of sodium hypochlorite salt (NaOCl) that comes from a mixture of HOCl and NaOH (partially hydrolyzed), if the Ka HOCl is 10^−5^ and there is an increase in the pH of the solution mixture.	3	
Water purification with alum KAl(SO₄)₂·12H₂O is the concept of salt hydrolysis, formed of H_2_SO_4_ and Al(OH)_3_.	7	Determining aluminum salt (Al_2_(SO_4_)_3_) properties in the water	1	
	8	Determining aluminum salt (Al_2_(SO_4_)_3_) properties in the water that is partially hydrolyzed by the Al^3+^ ion	1	
The sodium tripolyphosphate (STPP) in detergents can pollute the environment, a eutrophication process.	9	Determining the properties of detergent solution causing eutrophication	1	
	10	Determining the properties of detergent solution (sodium tripolyphosphate salt) that is partially hydrolyzed	1	
	11	Determining the impact of the disposal of detergent waste on the environment	2	
ZA fertilizer (NH4)_2_SO_4_ is an acidic salt.	12	Determining the properties of ammonium sulfate salt (NH4)_2_SO_4_	1	
	13	Stating the equation of (NH_4_)_2_SO_4_ reaction in the water, partially hydrolyzed	2	
Monosodium glutamate (C_5_H_8_NO_4_Na) is L-glutamic acid salt, adversely impactful on human health	14	Students' attitude towards the use of monosodium glutamate (C_5_H_8_NO_4_Na)	2	
	15	Determining the properties of monosodium glutamate salt (C_5_H_8_NO_4_Na)	1	

Each item was designed in three questions (Q1, Q2, Q3) that integrated diagnostic [68, 69] and summative measurements [10] and certainty of response index (CRI) [70, 71]. Students' responses to items (Q1, Q2, Q3) were evaluated based on the rubric (Table 2). For example, students' responses to items were as follows: Q1, Q2 “correct”, and Q3 “very sure” under the code CCC. Such a code indicated that students' conceptual understanding was in level 6, category of Scientific Knowledge (SK). On the other hand, if the response patterns in Q1, Q2 “incorrect” and Q3 “not sure”, the code would be IIU, implying that students' conceptual understanding was in the category of Lack of Knowledge (LOK), or level 1. This instrument had been validated from the aspects of item conformity with the construct variable and language. The validity results by three experts were stated under Fleiss’ kappa (K = .96), meaning that the experts agreed that the item validity was categorized good.

**Table 2:** All possibilities of responses [70, 71, 72].

(Q1)	(Q2)	(Q3)	Code	Conceptual Understanding Category	Level
Correct	Correct	Certain	CCC	Scientific Knowledge (SK)	6
Correct	Incorrect	Certain	CIC	Misconception False Positive (MFP)	5
Incorrect	Correct	Certain	ICC	Misconception False Negative (MFN)	4
Incorrect	Incorrect	Certain	IIC	All-Misconception (ALM)	3
Correct	Correct	Uncertain	CCU	Lack of Confidence/Lucky Guess. (LG)	2
Correct	Incorrect	Uncertain	CIU	Lack of Knowledge (LOK)	1
Incorrect	Correct	Uncertain	ICU	Lack of Knowledge (LOK)	1
Incorrect	Incorrect	Uncertain	IIU	Lack of Knowledge (LOK)	1

### 2.4 Data collection and analysis

Before the intervention, this research underwent pre-test data collection; whereas, the post-test data collection was done after the intervention. The construction of pre- and post-test items was the same. Students wrote down their responses on the provided answer sheet. Both tests were supervised by teachers in the school. The students must work on all items according to the allocated time (45 min). The instrument was immediately collected and should have the same number as the total participants.

The pre- and post-test measurement data were still ordinal data. The Rasch Partial Credit Model with WINSTEPS 4.5.5 software [27, 73] was used to convert ordinal data into interval data to have the same logit scale. The result was a data calibration of the levels of student's ability and item difficulty in the same interval.

The stacking analysis technique put pre-test and post-test data vertically [74]; meanwhile, the items appeared once in the experimental and control groups, allowing the researchers to check out any changes of the students after the intervention [56]. The examination was based on the same item, making the changes in students’ ability during the pre- and post-test be measured [56]. Hence, each student created two measures of abilities, namely pre-test and post-test, and one measure for each item. The research hypothesis is that the students' conceptual understanding from pre-test to post-test changes, both in the experimental and control groups.

Conversely, the racking analysis technique put both pre- and post-test data horizontally, in which each item appeared twice in data collection, and students' ability only emerged once. This enabled the researchers to check out the effects of learning implementation on each student's ability from the tests, especially the changes in item difficulty levels before and after the intervention [56].

## 3 Results

### 3.1 Rasch analysis properties of instrument

The summary of changes in concepts and items analyzed by the Rasch model is presented in Table 1. Table 2 provides the item fit statistic. An item is considered to experience a misfit if the measurement result is not in line with the following three criteria: Outfit mean-square residual (MNSQ): .5 < y < 1.5; Outfit standardized mean-square residual (ZSTD): -2 < Z < +2; and point measure correlation (PTMEA CORR): .4 < x < .8 [25]. All items comply with the Outfit MNSQ criterion; item 15 does not meet the Outfit MNSQ criterion; five items (item 1, 6, 12, 13, and 15) are not in accordance with the Outfit (ZSTD) criterion; all items meet the PTMEA CORR criterion. Simply put, all items fulfill those criteria mentioned previously (none having a misfit), and are fit and valid.

This instrument has a good unidimensionality (Appendix 1). Raw variant index arrives at above the standard of 20% (33.9%), indicating that the instrument can effectively measure students' understanding of the hydrolysis concept [75]. The assessment scale analysis (Appendix 2) informs that the observation mean starts from logit -1.73 for category 1 (LOK) to logit +1.76 (category 6, SK). This signifies that the category of students’ understanding takes place consistently [27]. In addition, the high item separation index (logit 6.71) and the high item reliability (logit .98) (Table 3) indicate that the respondents (students) are sufficient to confirm the level of item difficulty, strengthening the instrument construct validity [27]. The higher the item separation and reliability index, the more confident the researchers are about replicating item placement in other suitable sample students [25, 27]. Person separation index and person reliability that reach logit 2.0 and logit .75 (Table 4), respectively, imply that the instrument is quite sensitive to differentiate the high and low abilities of the students [25, 27]. According to the Rasch model calculation, the coefficient of Cronbach Alpha of logit .81 (Table 4) reflects an interaction between 380 students and 15 items with an excellent category [24, 76]. In other words, the interaction between students and items is very significant. The instrument has an excellent internal psychometric consistency and is considered very reliable.

**Table 3:** Item statistics: misfit order.

Item	Difficult	Error	Outfit MNSQ	Outfit ZSTD	PTMEA CORR.
1	-.38	.05	1.36	2.87	.47
2	.20	.04	1.13	1.56	.49
3	-.36	.05	.91	-.79	.43
4	.33	.04	1.09	.77	.55
5	-.25	.05	.94	-.55	.56
6	.26	.04	1.20	2.44	.41
7	.15	.04	.91	-1.17	.54
8	.47	.04	.90	-1.45	.44
9	-.47	.05	1.19	1.49	.46
10	.08	.04	1.09	1.04	.55
11	-.34	.05	1.04	.42	.51
12	-.06	.04	.71	-3.50	.60
13	.46	.04	.74	-4.12	.55
14	-.36	.05	1.00	.77	.55
15	.26	.04	1.31	3.74	.47

**Table 4:** Person separation and reliability statistics.

Parameter	Measure	SD	Separation	Reliability	INFIT		OUTFIT		KR-20
					MNSQ	ZSTD	MNSQ	ZSTD	
Person (N = 380)	.67	.52	1.72	.75	1.00	.04	1.02	.10	.81
Item (N = 15)	.00	.32	6.71	.98	1.07	.41	1.02	-.01	

### 3.2 The difference in students’ understanding ability of hydrolysis concept

The result of the Mann-Whitney test (Table 5) brings out the fact that statistically, there is a significant difference in the results of pre-test (U = 3459.000), p < 0.05) and post-test (U = 1723.000, p < 0.05) among students in experimental and control groups. Further, the Wilcoxon test result (Table 6) shows that the results of pre-test and post-test of students in the experimental group (Z = -8.076) and the control group (Z = -6.690) at the significant level *(p)* < 0.05 are significantly different. This suggests that students’ understanding of the hydrolysis concept after the intervention (post-test) is higher than before the intervention (pre-test), both in experimental and control groups. However, the abilities of students in the experimental group are better than those in the control group. Accordingly, the learning process with the POGIL in the SSI context is better than the conventional learning.

**Table 5:** The result of the Mann-Whitney U test based on students’ pre-test and post-test abilities in experimental and control groups (p < 0.05).

Test	Experimental Group (N = 97)	Control Group (N = 93)	U	p
Pre-test	0.5026 (-0.57–1.26)^a^	0.3029 (-1.61–1.03)^a^	3459.000	0.005
Post-test	1.1722 (-0.09–3.00)^a^	0.7052 (-1.06–1.47)^a^	1723.000	0.000

**Table 6:** The result of the Wilcoxon test of students’ pre-test and post-test in experimental and control groups (p < 0.05).

Group	Pre-test	Post-test	Z	p∗
Experimental	0.5026 (-0.57–1.26)^a^	1.1722 (-0.09–3.00)^a^	-8.076	0.000
Control	0.3029 (-1.61–1.03)^a^	0.7052 (-1.06–1.47)^a^	-6.690	0.000

### 3.3 The changes in students’ understanding ability of the hydrolysis concept

From the different changes in pre- and post-test (Table 7), students in the experimental and control groups have improved their understanding of the hydrolysis concept. The experimental group's mean of pre-test and post-test is logit .51 (S.E = logit .21) and logit 1.50 (S.E = logit .32), respectively, with the mean difference of both tests is (logit .99). In contrast, the mean of pre-test and post-test of the control group gets logit .26 (S.E = logit .20) and logit .87 (S.E = logit .26), respectively, with the mean difference of pre- and post-test is logit .61. Such differences indicate different effects of interventions in the experimental and control group.

**Table 7:** Logit of mean of pre- and post-test items of experimental and control groups.

Group	Student	Item	Mean/SE (logit)		
			Pre-test	Post-test	Pre- and Post-test Difference
Experimental	97	15	.51/(.21)	1.50/(.32)	.99
Control	93	15	.26/(.20)	.87/(.24)	.61

Description: SE = Standard Error.

**Figure 1:**
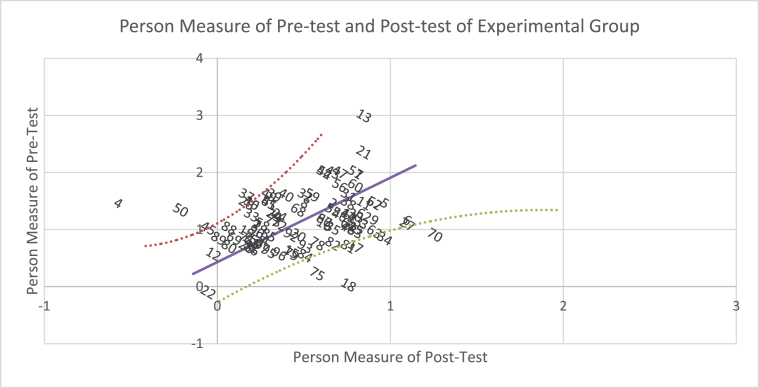
Scatter plots of person measures in pre- and post-test of the experimental group.

**Figure 2:**
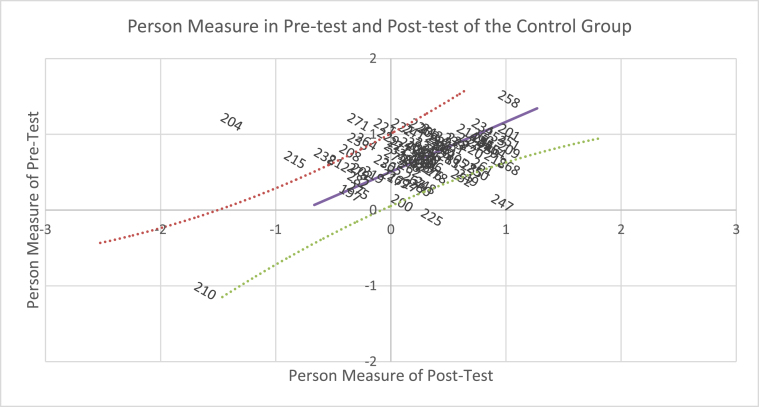
Scatter plots of person measures in pre- and post-test of the control group.

If the pre-test and post-test results of the experimental group are plotted in pairs (Figure 1), so that the mean difference in the sample pre- and post-test (logit +.99) is displayed as an intercept on the horizontal axis with the plotted slope = 1, several facts obtained: First, two lines that form the upper and lower curves separate 66 students around the empirical plot line, in which the pre-test and post-test mean is not significantly different from the mean difference in the pre- and post-test in the experimental group. Second, above the curve, 23 students experience significant changes; the mean of pre- and post-test is greater than the mean difference in sample pre-test and post-test. Third, seven students do not change, and ten students have negative changes (under the curve), so that they are under the curve. Similarly, the results of pre- and post-test of the control group (Figure 2) show that 53 students are around the empirical plot line; the abilities of 25 students change significantly (greater than the mean of sample pre- and post-test (logit +.61); two students do not change; 13 students experience negative changes in abilities. The difference in the plotting of pre-test and post-test results signifies different effects of interventions in the experimental and control groups.

### 3.4 The changes in item difficulty level

Table 8 presents the results of the racking analysis in connection with the changes in item difficulty level in the pre- and post-test of experimental and control groups. It is shown that in terms of item difficulty level, the mean of pre-test of the experimental group is (logit .32), the mean of post-test is (logit -.34), and the mean difference of the pre- and post-test is (logit -.66). Moreover, the mean of pre-test of the control group is (logit .25), the mean of post-test is (logit -.25), and the mean difference of the pre- and post-test is (logit -.50). This research also finds out that seven items have significant changes in the item difficulty level in the experimental group, lower than the pre- and post-test mean difference of (logit -.66), namely item 1, 2, 5, 7, 9, and 11. Eight items with a difficulty level greater than the mean are item 3, 4, 6, 8, 12, 13, 14, and 15. Item 10 has the same difficulty level as the mean. In the control group, eight items change significantly or less than the pre- and post-test mean difference of (logit -.50), including item 2, 3, 4, 5, 9, 11, 12, and 14; five items (item 1, 6, 7, 8, 10, 13) are greater than the mean; one item (item 15) has negative changes or becomes more difficult. The most difficult item in the experimental group is item 1 (.80 logit) and the easiest one is item 14 (logit -.10). Meanwhile, the most difficult item in the control group is item 13 (logit .64), and item 3 (logit -.15) is the easiest one. These findings indicate differences in the item difficulty level changes between students taught by the POGIL in the SSI context and the conventional model.

**Table 8:** Data of item measures of pre- and post-test of experimental and control groups.

Item	Experimental (Mean)			Control (Mean)		
	Pre-test	Post-test	Difference Pre- and Post-test	Pre-test	Post-test	Difference Pre- and Post-test
Item1	.16	-1.00	-1.16	-.06	-.76	-.7
Item2	.80	.01	-.79	.39	-.40	-.79
Item3	.20	-.63	-.43	-.15	-.83	-.68
Item4	.62	.25	-.37	.54	.02	-.52
Item5	.14	-.78	-.92	.10	-.49	-.59
Item6	.26	.22	-.04	.41	.30	-.11
Item7	.66	-.33	-.99	.33	-.06	-.39
Item8	.59	.45	-.14	.49	.47	-.02
Item9	-.04	-.85	-.81	-.08	-.93	-.85
Item10	.40	-.26	-.66	.32	-.01	-.33
Item11	.13	-.91	-1.04	.05	-.78	-.83
Item12	.33	-.23	-.56	.25	-.51	-.76
Item13	.77	.16	-.61	.64	.33	-.31
Item14	-.10	-.80	-.7	.15	-.83	-.98
Item15	.25	-.40	-.65	.39	.72	.33
Mean	.32	-.34	-.66	.25	-.25	-.50

### 3.5 Conceptual changes in students’ ability and item difficulty levels

Apart from the effect of learning interventions, there are three other factors that tend to influence the changes in students' ability and item difficulty levels, as follows: 1) guessing which happened to be correct or (lucky guess), 2) cheating, 3) carelessness. These factors can be identified from the student's item response pattern using a scalogram. For instance, the response pattern of post-test item 7 for student 353, 375, and 170 (Table 9). These three students, in the seventh and eighth row from the left, cannot understand item 12 (logit -.06) and item 10 (logit .08). Meanwhile, they can correctly explain the more difficult item, i.e., item 7 (logit .15). This situation implies a lucky guess, which in fact, these students have higher post-test abilities than the item 7 logit. Next is a cheating indication in the response pattern of student 128, 129, 134, 137, and 146. Such an indication is initially detected from the same post-test mean (logit 1.61) and item response pattern. The last one is carelessness, e.g., student 110, 118, and 139 are considered to be careless as they cannot correctly explain the easy item 4 (logit .33), yet can accurately understand item 13 (logit .46), which is harder than item 4. Moreover, they get very high post-test abilities.

**Table 9:**
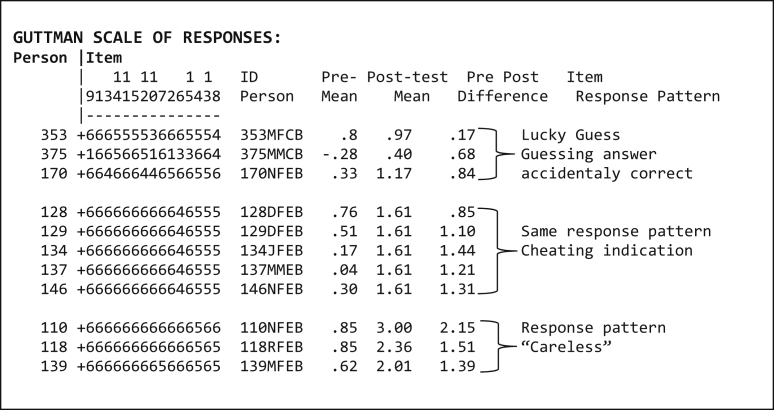
Scalogram.

### 3.6 Negative changes

Negative changes in conceptual understanding are detected from the changes in students’ post-test logit less than the pre-test logit. For example, two students from the experimental group (E18 and E75) and the control group (C225 and C247) are taken; they have negative changes (Table 10). This means that these four students experience decreased abilities after the intervention. The pre-test item mean and the post-item mean of student E18 are (logit .76) and (logit .04), sequentially, with the mean difference of pre- and post-test arriving at (logit -.72). Moreover, the pre- and post-test item standard errors of student E18 are (logit .22) and (logit .18), respectively, with the combined standard error of logit .40. On account of the higher combined standard error than the pre- and post-test measures, the ability of student E18 in both tests is not significantly different. This also applied to student E75, C225, and C247.

**Table 10:**
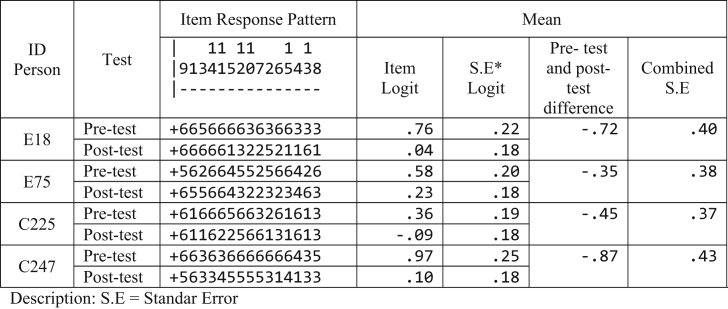
Scalogram results of student E18, E75, C225, and 247.

## 4 Discussion and conclusion

The findings show changes in students' understanding abilities of the hydrolysis concept and items after the intervention. From the pre- and post-test mean difference, the experimental group has better positive changes than the control group [58]. In addition to the effect of the intervention, there is another factor contributing to the positive conceptual changes mentioned above, in terms of students’ ability and item difficulty levels [24, 58]. The factor refers to some students who “accidently” give a correct response pattern (in the post-test). Even so, both groups have also experienced negative changes, implying that the intervention is specifically responded by students on account of the carelessness factor or a misconception-comprising response pattern [56, 58, 77]. Regarding this, not all learning objectives of the hydrolysis concept match the approach of POGIL in the SSI context. Negative changes of the students are because they are not epistemologically involved in the learning process, particularly in the observing, measuring, and calculating stages. These activities are interrelated up to group discussions as part of the stages of conceptual formation based on empirical facts [78]. Students are expected to explain and link the concepts they have learned following their epistemological reasoning [16, 79].

Furthermore, the interpretation of changes due to pedagogical interventions is exemplified by four students (Table 8) in item 5. In the pre-test, the ability of student E18 (logit .76), student E75 (logit .58), student C225 (logit .36), and student C247 (logit .96) is greater. They also respond to item 5 (-.25 logit) accurately. However, in the post-test item 5, the response of student E18, E75, C225, and C247 is incorrect due to their decreased post-test abilities. Therefore, the pre- and post-test mean difference is lower than item 5. Why do these changes occur? Such changes are exemplified by the response pattern of student E18 in item 5. This item measures students’ ability in determining the reaction of NaOCl reaction: ${\text{OCl}}^{-}+\;{\text{H}}_{2}\text{O}\to \text{HOCl}+\;{\text{OH}}^{-}$, with the estimated pH = 7 and is alkaline. The question (Q1) of this item is, “is it correct that NaOCl is alkaline?“. E18 answers “correct” in the pre-test, yet responds to “incorrect” in the post-test. The question (Q2) of this item is “what is your consideration for your answer in the Q1?“. Four options are provided: (a) because NaOCl is formed of strong acids and weak bases; (b) because NaOCl is formed of weak acids and strong bases; (c) because NaOCl is formed of weak acids and weak bases; (d) because NaOCl is formed of strong acids and strong bases. In the pre-test, E18 chooses the correct answer (b), yet selects the incorrect answer (a) in the post-test that comprises misconception. Next, in the Q3 of this item, E18 chooses “very sure” in the pre-test and “not sure” in the post-test. The item 5 response pattern of E18 becomes CCC (category of scientific knowledge - SK) in the pre-test and IIU (category of lack of knowledge - LOK) in the post-test. Accordingly, the response pattern changes from CCC to IIU. The pre- and post-test mean difference of E18 (logit -.72) lower than item 5 (-.25) signifies that the error of response pattern results from misconception. This also applies to the response pattern of E75 (logit -.35), C225 (logit -.45), and C247 (logit -.87).

The misconception refers to the inability to identify the NaOCl salt hydrolysis that is formed of weak acids and strong bases. In short, the four students tend to not understand the concept of acid and base and acid-base reaction. These findings strengthen several previous studies [51, 53, 54, 80]. A study on the understanding of the acid-base concept of senior high school students in Malaysia concludes that some students have little understanding of the function of detergents as the cleaning agent, the difference between strong acids and strong bases, and the treatment for soil acidity using fertilizers [53]. In the same tune, such little understanding is because they do not conceptualize acid-base strength as a property that arises from the interaction of many reaction factors [51]. Additionally, research on an alternative conception of salt hydrolysis among senior high school students contends that the concept of hydrolysis is challenging for the students [54]. They are usually able to state the acidity of a salt solution correctly, yet writing a chemical equation to explain such a phenomenon is a great challenge. Most of the alternative conceptions are identifiable, rooted in the misunderstanding of equilibrium process, acid and base, material structure and other basic problems, student tendency to use a wrong analogy, and the lack of laboratory practice.

This research findings and elaboration of negative changes (case E18) prove the advantages of the Rasch model, specifically its potential in linking the result of changes (pre- and post-test), the item difficulty level, and the content being measured [18]. Such information solely comes from the Rasch model-based stacking and racking analysis techniques. The stacking technique provides information regarding “who has changed”; in contrast, the racking technique offers information of “what has changed” [56, 58], allowing the researchers to spell out the effect of the applied pedagogical innovation [18, 33, 34]. Although the instrument measurement result of this work is not data-rich, the analysis strength of the Rasch model can describe in detail the conceptual changes, both in the students’ ability and item difficulty levels.

### 4.1 Limitations and further studies

The primary limitation of this research is that it did not take into account the aspects of learning style, culture, and motivation that can change due to learning interventions. Future studies, therefore, can address these aspects. The present study can be continued by considering the context of a problem that closely connects with the parameter of item difficulty level. The analysis will be more interesting if it can prove that different item difficulty levels are influenced by problem contexts in each item [81]. Further studies are also expected to find an analysis technique that can integrate problem contexts, item characteristics, and item difficulty levels in a measurement model. It is assumed that different problem contexts in each item will be more likely to affect measurement results because problem contexts have conceptual linkage with items and student activities in doing experiments, measuring, interpreting data/graphs, and others. Thus, the linkages between the learning process during the intervention and conceptual changes in students’ ability and item difficulty levels can be explained in detail; which part of the process leads the students to change their understanding related to specific ideas taught to them.

## Declarations

### Author contribution statement

Lukman Abdul Rauf Laliyo: Conceived and designed the experiments; Performed the experiments; Analyzed and interpreted the data; Contributed reagents, materials, analysis tools or data; Wrote the paper.

Bambang Sumintono: Analyzed and interpreted the data; Wrote the paper.

Citra Panigoro: Contributed reagents, materials, analysis tools or data; Wrote the paper.

### Funding statement

This research did not receive any specific grant from funding agencies in the public, commercial, or not-for-profit sectors.

### Data availability statement

Data included in article/supplementary material/referenced in article.

### Declaration of interests statement

The authors declare no conflict of interest.

### Additional information

No additional information is available for this paper.

## Figures and Tables

**Figure:**
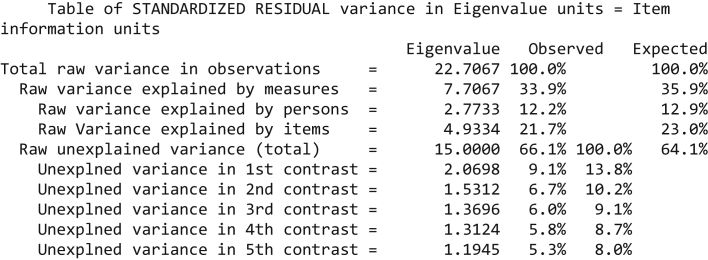


**Figure:**
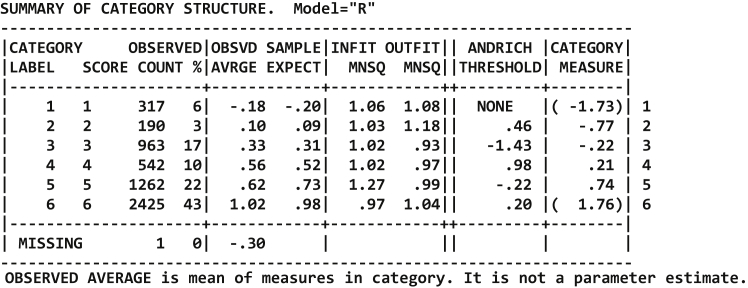


## Appendix

Appendix 1. Standardized residual variance in eigen value units.

Appendix 2. Summary of category structure.
